# Etiology and Antimicrobial Resistance of Culture-Positive Infections in Ugandan Infants: A Cohort Study of 7000 Neonates and Infants

**DOI:** 10.1093/ofid/ofae629

**Published:** 2025-03-10

**Authors:** Hannah G Davies, Mary Kyohere, Valerie Tusubira, Alexander Amone, Amusa Wamawobe, Cleophas Komugisha, Philippa Musoke, Lauren Hookham, Pooja Ravji, Melanie Etti, Juliet Nsimire Sendagala, Dan R Shelley, Caitlin Farley, Merryn Voysey, Owen B Spiller, Joseph Peacock, Musa Sekikubo, Paul T Heath, Kirsty Le Doare, PROGRESS Study Author Group, Abdelmajid Djennad, Agnes Nyamaizi, Agnes Ssali, Alexander Amone, Amusa Wamawobe, Annettee Nakimuli, Caitlin Farley, Carol Nanyunja, Christine Najuka, Cleophas Komugisha, Dan R Shelley, Edward A R Portal, Ellie Duckworth, Emilie Karafillakis, Geraldine O’Hara, Godfrey Matovu, Hannah G Davies, Janet Seeley, Joseph Peacock, Juliet Nsimire Sendagala, Katie Cowie, Kirsty Le Doare, Konstantinos Karampatsas, Lauren Hookham, Madeleine Cochet, Margaret Sewegaba, Mary Kyohere, Maxensia Owor, Melanie Etti, Merryn Voysey, Moses Musooko, Musa Sekikubo, Owen B Spiller, Patience Atuhaire, Paul T Heath, Philippa Musoke, Phiona Nalubega, Pooja Ravji, Richard Katungye, Ritah Namugumya, Rosalin Parks, Rose Azuba, Sam Kipyeko, Simon Beach, Stephen Bentley, Tim Old, Tobius Mutabazi, Valerie Tusubira, Vicki Chalker

**Affiliations:** Institute for Infection and Immunity, St George's, University of London, London, UK; Makerere University—Johns Hopkins University (MUJHU) Research Collaboration, Kampala, Uganda; Department of Clinical Research, Faculty of Infectious and Tropical Diseases, London School of Hygiene & Tropical Medicine, London, UK; Institute for Infection and Immunity, St George's, University of London, London, UK; Makerere University—Johns Hopkins University (MUJHU) Research Collaboration, Kampala, Uganda; Makerere University—Johns Hopkins University (MUJHU) Research Collaboration, Kampala, Uganda; Makerere University—Johns Hopkins University (MUJHU) Research Collaboration, Kampala, Uganda; Department of Medical Microbiology, Makerere University, Kampala, Uganda; Makerere University—Johns Hopkins University (MUJHU) Research Collaboration, Kampala, Uganda; Makerere University—Johns Hopkins University (MUJHU) Research Collaboration, Kampala, Uganda; Institute for Infection and Immunity, St George's, University of London, London, UK; Makerere University—Johns Hopkins University (MUJHU) Research Collaboration, Kampala, Uganda; Medical Research Council/Uganda Virus Research Institute and London School of Hygiene & Tropical Medicine Uganda Research Unit, Entebbe, Uganda; Department of Infectious Diseases, Cambridge University Hospitals NHS Foundation Trust, Cambridge, UK; Institute for Infection and Immunity, St George's, University of London, London, UK; Makerere University—Johns Hopkins University (MUJHU) Research Collaboration, Kampala, Uganda; Medical Research Council/Uganda Virus Research Institute and London School of Hygiene & Tropical Medicine Uganda Research Unit, Entebbe, Uganda; Cardiff University School of Medicine, Division of Infection & Immunity, Cardiff, UK; Cardiff University School of Medicine, Division of Infection & Immunity, Cardiff, UK; Oxford Vaccine Group, Department of Paediatrics, University of Oxford, Oxford, UK; Cardiff University School of Medicine, Division of Infection & Immunity, Cardiff, UK; Institute for Infection and Immunity, St George's, University of London, London, UK; Department of Obstetrics and Gynaecology, Makerere University, Kampala, Uganda; Institute for Infection and Immunity, St George's, University of London, London, UK; Institute for Infection and Immunity, St George's, University of London, London, UK; Makerere University—Johns Hopkins University (MUJHU) Research Collaboration, Kampala, Uganda; Medical Research Council/Uganda Virus Research Institute and London School of Hygiene & Tropical Medicine Uganda Research Unit, Entebbe, Uganda

**Keywords:** antimicrobial resistance, bloodstream infections, neonatal sepsis, respiratory infections, surveillance

## Abstract

**Background:**

Epidemiological evidence about the etiology and antimicrobial resistance of neonatal infections remains limited in low-resource settings. We aimed to describe the etiology of neonatal infections in a prospective observational cohort study conducted at two hospital sites in Kampala, Uganda.

**Methods:**

Babies admitted to either unit with risk factors or signs of sepsis, pneumonia, or meningitis had a blood culture, nasopharyngeal swab, and lumbar puncture (if indicated) collected. Basic demographics were collected, and babies were followed up until discharge or death to determine admission outcome. Blood cultures were processed using the BACTEC system and identification confirmed by matrix-assisted laser desorption/ionization time-of-flight mass spectrometry. Cerebrospinal fluid was processed using standard microbiological testing and swabs were processed using the multiplex real-time polymerase chain reaction assay. Antimicrobial susceptibilities of bacterial isolates to World Health Organization–recommended first-line antibiotics (ampicillin or benzylpenicillin and gentamicin) were assessed using e-tests.

**Results:**

A total of 7323 infants with signs or risk factors for sepsis had blood cultures, 2563 had nasopharyngeal swabs, and 23 had lumbar punctures collected. Eleven percent of blood cultures and 8.6% of swabs were positive. Inpatient mortality was 12.1%, with 27.7% case fatality observed among infants with Gram-negative bloodstream infections. *Escherichia coli* (14.8%), *Acinetobacter* spp. (10.3%), and *Klebsiella* spp. (7.6%), were notable contributors to Gram-negative sepsis, whereas Group B *Streptococcus* was the predominant Gram-positive pathogen identified (13.5%). Almost 60% of Gram-negative pathogens were ampicillin- and gentamicin-resistant.

**Conclusions:**

Our study demonstrates high levels of antimicrobial resistance and inpatient mortality from neonatal sepsis in the first months of life in Uganda. This underscores the pressing need for revised, context-specific antimicrobial treatment guidelines that account for the evolving landscape of antimicrobial resistance in neonatal sepsis.

## Teaser

Nested within a prospective case control study, in this cohort study we describe the etiology of neonatal infections and patterns of antimicrobial resistance in two hospitals in Kampala, Uganda, demonstrating high levels of antimicrobial resistance and inpatient mortality in the first months of life in this setting.

Globally, infectious diseases remain a leading cause of death in children younger than five years of age, with the largest proportion occurring in neonates and infants. The first months of a child's life are the most vulnerable with 47% of all under-5 deaths occurring in the first 28 days of life [1]. The burden is unevenly distributed globally with the highest rates occurring in sub-Saharan Africa, which reported an estimated 27 neonatal deaths per 1000 live births in 2020 [1, 2]. Despite a reduction of 24% in the past 20 years, this is still significantly more than the Sustainable Development Goal target of fewer than 12 neonatal deaths per 1000 live births [1]. An estimated 11% are caused by infections [2]. The most common infectious causes of death in the neonatal period are lower respiratory infections (3.8%) and sepsis or meningitis (3.7%) [3].

Blood culture remains the gold standard for identification of infective organisms in neonatal sepsis but is costly and not always available in settings with few resources. Admitted neonates are started on antibiotics empirically because of the need for timely intervention to prevent deterioration and death. The World Health Organization (WHO) Pocket Book of Hospital Care for Children recommends intramuscular or intravenous antibiotic therapy with a combination of gentamicin and benzylpenicillin or ampicillin for at least 7–10 days in neonates younger than 28 days who fulfill the case definition of serious bacterial infection, with a second-generation cephalosporin as second-line treatment [4]. However, there has been concern regarding the emergence of resistance to these antibiotics [5, 6].

Recent studies from low- and middle-income countries (LMICs) have shown an emerging preponderance of Gram-negative sepsis and concerning increases in resistance rates to the WHO first- and second-line recommended antibiotics [5]. It has been estimated that 214 000 neonatal deaths globally are attributable to resistant pathogens [7]. Despite growing awareness of the importance of antimicrobial resistance (AMR), epidemiological evidence remains limited and available data are not sufficient to draw a true, recent, or accurate picture of antibiotic resistance among neonates in many LMICs [8].

Respiratory viral infections such as respiratory syncytial virus are increasingly recognized as a cause of morbidity and mortality in young infants in Africa. However, viral infections frequently go undiagnosed or are identified late during an infectious episode. As improved, low-cost diagnostics for viral pathogens have emerged, it is now possible to better understand their significance in infant infectious disease admissions.

In 2020, Uganda had a neonatal mortality rate of 19.2 per 1000 live births, with neonatal deaths accounting for 60% of all deaths in the first year of life [9]. Despite these high numbers, data on specific infectious causes of neonatal morbidity and mortality are lacking, as are data on AMR patterns. This study therefore aimed to identify the common bacterial and viral causes of young infant sepsis, lower respiratory tract infections, and meningitis including antimicrobial resistance patterns in two tertiary Ugandan hospitals and to describe factors associated with inpatient mortality.

## METHODS

### Study Design

This paper forms part of a supplement based on the PROGRESS study. The Progressing Group B Streptococcal Vaccines (PROGRESS) study aimed to describe the causes of infectious mortality and morbidity in pregnancy and neonates, as well as the seroepidemiology of Group B Streptococcal infection—the major cause of neonatal sepsis worldwide—in Kampala, Uganda. Detailed information regarding the PROGRESS research protocol has been published separately [10]. Supplementary Figure 1 outlines the participant recruitment sites for the studies that form part of this supplement.

This prospective observational cohort study was conducted between 24 April 2019 and 31 December 2020 in two hospitals in Kampala, Uganda: Mulago National Referral Hospital (MNRH) and Kawempe National Referral Hospital (KNRH).

### Participants, Inclusion and Definitions

This was a prospective observational cohort study of infants presenting with signs or risk factors for infection (sepsis, pneumonia, or meningitis) that aimed to describe the etiology of bacterial and viral admissions in young infants, antimicrobial resistance in those with bloodstream infections, and factors associated with in-hospital mortality. Eligible participants included neonates and young infants (up to 90 days) presenting with signs of infection (sepsis, meningitis, or pneumonia). Details of the signs and symptoms sought can be found in Table 1. Any eligible infant aged 0–90 days presenting to either of the study hospitals’ neonatal and pediatric units with signs of infection or at least two risk factors for sepsis had a blood culture, lumbar puncture where indicated, and nasopharyngeal swab (NPS) collected as part of their routine care. Samples were collected before receipt of antibiotics where possible. A positive blood culture was defined as positive for bacterial growth (see laboratory methods for details). Bacteria were then defined as likely pathogens or likely contaminants based on the expertise of a panel of microbiologists and infectious disease physicians in the United Kingdom and Uganda (Supplementary Table 1 outlines a full list of contaminants). Early onset sepsis was defined as sepsis between days zero and six and late onset sepsis was defined as sepsis between days seven and 90.

**Table 1.** Demographics and Clinical Characteristics of the 7323 Infants Enrolled

Characteristics	Number (%)
Principal reason for admission (n = 7050)	
Suspected sepsis/infection	2104 (29.8)
Prematurity	2295 (32.6)
Birth asphyxia	2072 (29.4)
Birth defect	48 (0.7)
Other	531 (7.5)
Infant sex (n = 6469)	
Male	3610 (55.8)
Female	2859 (44.2)
HIV exposure (n = 4492)	
Exposed	460 (10.2)
Unexposed	4032 (89.8)
Gestational age at birth (n = 5570)	
Term	3323 (59.7)
Preterm	2247 (40.3)
Outcome of admission (n = 5960)	
Died	721 (12.1)
Discharged home alive	5156 (86.5)
Transferred to another facility	83 (1.4)
Low birthweight at birth (n = 3452)	
<2500 g	1492 (43.2)
≥2500 g	1960 (56.8)
Low weight on admission (n = 4470)	
<2500 g	1870 (41.8)
≥ 2500 g	2600 (58.2)
Age at admission (n = 6467)	
0 d	4315 (66.7)
1–6 d	1599 (24.7)
7–28 d	339 (5.2)
>28 d	214 (3.3)
Mode of delivery (n = 5496)	
Vaginal	3539 (64.4)
Cesarean section	1957 (35.6)
Numbers of signs of sepsis at admission (n = 7323)	
Risk factors only	993 (13.6)
1–3 signs	4713 (64.4)
>3 signs	1617 (22.1)
Signs and symptoms by system (n = 7323)^a^	
Respiratory signs	4480 (61.2)
Neurological signs	1029 (14.1)
Cardiovascular signs	282 (3.9)
Gastrointestinal signs	2888 (39.4)
Skin signs	495 (6.7)
Fever, hypothermia or temperature instability	3204 (43.8)
Other signs	105 (1.4)
No signs and symptoms (risk factors only)	993 (13.6)

^a^Cardiovascular signs—delayed capillary refill time >3 seconds, abnormal heartrate (>180 bpm or <100).

Gastrointestinal signs—difficulty feeding or feed intolerance, abdominal distension, diarrhoea, vomiting, signs of dehydration.

Skin signs—jaundice, multiple or severe skin pustules, pus from the umbilical stump, petechial rash.

Respiratory signs—chest indrawing, grunting, cyanosis, apnea, oxygen requirement.

Neurological signs—seizures, hypotonia, irritability, lethargy, bulging fontanelle, no movement or movement only when stimulated.

### Clinical Sampling

Collection of blood and cerebrospinal fluid for culture occurred between April 2019 and December 2020. Consent was sought for a flocked nasopharyngeal swab (Copan 484CE), lumbar puncture if appropriate, and 1–2 mL of blood for culture. A smaller sample was collected from very low birthweight babies, according to their birthweight (Supplementary Table 2). The volume of blood taken was documented on the sample request form and blood culture bottles were weighed before and after sample to check volumes. Blood was inoculated into BD BACTEC Peds Plus bottles and then sent for culture at Makerere University Clinical Microbiology Laboratory (MUCML). MUCML has been accredited by the College of American Pathologists for microbiological testing since 2018. Transport was arranged twice daily from the study sites to the Clinical and Laboratory Standards Institute–accredited laboratory, located within the central Kampala region.

### Laboratory Methods

#### Blood Culture Processing

All blood cultures were incubated in an automatic BACTEC machine (BACTEC 9050,9120 Becton Dickinson, UK, or FX40 Becton Dickinson, USA). Samples that arrived at the laboratory after 12 hours of collection or those that showed a visible turbidity on arrival were assessed and subcultured before loading into the blood culture machine. All BACTEC-positive cultures were Gram stained and subcultured onto MacConkey, 5% Sheep Blood, and 5% Chocolate Blood agar (Biolab Hungary and Oxoid UK). MacConkey agar was incubated in ambient air while Sheep Blood and Chocolate Blood agar were incubated in 5% carbon dioxide at 37 °C. Samples that showed yeast cells or fungal elements in the Gram reaction were additionally subcultured on Sabouraud Chloramphenicol Agar. Plates were monitored every 24 hours for growth. Blood culture bottles were incubated for five days, at which point they were deemed negative. Bacterial isolates from positive culture were stored on cryo beads before shipment to the United Kingdom.

#### Identification of Organisms

Bacterial identification was based on the morphology, Gram stain, and standard biochemical tests for Gram-positive and Gram-negative identification. Positive blood cultures with an identified pathogen were shipped to St George's, University of London (SGUL) and organism identification confirmed with matrix-assisted laser desorption ionization time-of-flight mass spectrometry (MALDI-TOF) [11]. In the case of discrepant organism identification between SGUL and MUCML, the organism identified via MALDI-TOF was considered to be the identified organism. In instances where the isolate did not grow following shipment, the identification and antimicrobial susceptibility patterns from MUCML were presented. Organisms confirmed by MALDI-TOF were then shipped to Cardiff University for antimicrobial susceptibility testing.

#### Antimicrobial Susceptibility Testing

Single colonies from overnight culture on Colombia blood agar of frozen archived isolates were taken and resuspended to prepare 0.5 McFarland standards in sterile three mL 0.85% saline (Oxoid, UK) for antimicrobial sensitivity testing. Minimum inhibitory concentrations were determined for Gram-positive bacteria (benzylpenicillin, chloramphenicol, clindamycin, erythromycin, gentamicin, levofloxacin, tetracycline, and vancomycin). For Gram-negative bacteria, amikacin, amoxicillin-clavulanate, ampicillin, azithromycin, ceftazidime, chloramphenicol, ciprofloxacin, colistin, gentamicin, meropenem, tetracycline, and tigecycline were tested. Plates were inoculated using a multipoint inoculator (MAST URI DOT) post-autoclave sterilization of the pins. Inoculated plates were incubated at 37 °C for 18–24 hours. Susceptibility was determined according to the European Committee on Antimicrobial Susceptibility Testing [11] guidelines unless unavailable (where Epidemiological Cutoff Values or European Committee on Antimicrobial Susceptibility Testing determined pharmacokinetic/pharmacodynamic values were used).

#### Nasopharyngeal Swab Polymerase Chain Reaction

NPS were tested using the Allplex Respiratory Panel 4 (bacterial) and Respiratory Panel (viral) essential polymerase chain reaction assays at the Medical Research Council/Uganda Virus Research Institute and London School of Hygiene and Tropical Medicine laboratories in Entebbe, Uganda. Extraction was performed using the Quick-DNA/RNA Pathogen Miniprep DNA & RNA kit according to standard manufacturer's protocol. Amplification and detection were performed using BioRad CFX96TM Real-time PCR Detection System (CFX Manager Software-IVD v1.6). Data were analyzed in Seegene Viewer.

### Data Collection and Management

Relevant clinical information was extracted from the infant's hand-held or hospital notes onto the case report form in Research Electronic Data Capture (REDCap) data management platform [12] hosted at Makerere University—Johns Hopkins University. This included the mother's demographic details, infant's gestation at birth, delivery details, risk factors, signs and symptoms of infection, and outcome of admission. Early-onset sepsis was defined as infection in the first 6 days of life and late-onset from day seven onwards.

### Statistical Analysis

Sample size was determined by the parent PROGRESS study, which aimed to characterize incidence, serotype distribution and seroepidemiology of invasive Group B Streptococcus (GBS) disease [10]. We report means or medians and associated 95% confidence intervals (CI) or medians and interquartile ranges based on the distributions of the data. Multivariable logistic regression was performed to assess risk-factors associated with inpatient death. The data were visually inspected and cross-tabulations between each potential risk factor (preterm birth, maternal HIV status, age at admission, mode of delivery, bloodstream infection, number of signs of sepsis) and inpatient mortality were generated. Missing data, where present, were described. Odds ratios were examined to assess the association with mortality adjusting for each potential risk factor in turn, starting with the most strongly associated factor. The crude and adjusted odds ratios (OR) were examined for a change, with particular interest paid to a change of 10% or greater. Multicollinearity was assessed through use of Spearman rank correlation. Logistic regression was carried out in STATA (version 17.1). A subgroup analysis examining factors associated with case fatality limited to infants with confirmed bloodstream infections was also undertaken. STROBE checklists for observational studies were used to report enrolment flow (see Supplementary material).

## RESULTS

The characteristics of the 7323 infants that had blood cultures collected are presented in Table 1. More than half were male (55.8%) and the majority were born at full term (59.7%). Most infants had early-onset sepsis, with 66.7% admitted on their day of birth and 91.4% admitted within the first week. A total of 993 (13.6%) had risk factors for sepsis only, without clinical signs or symptoms, whereas 22.1% had greater than three signs of sepsis at admission.

**Figure 1.**
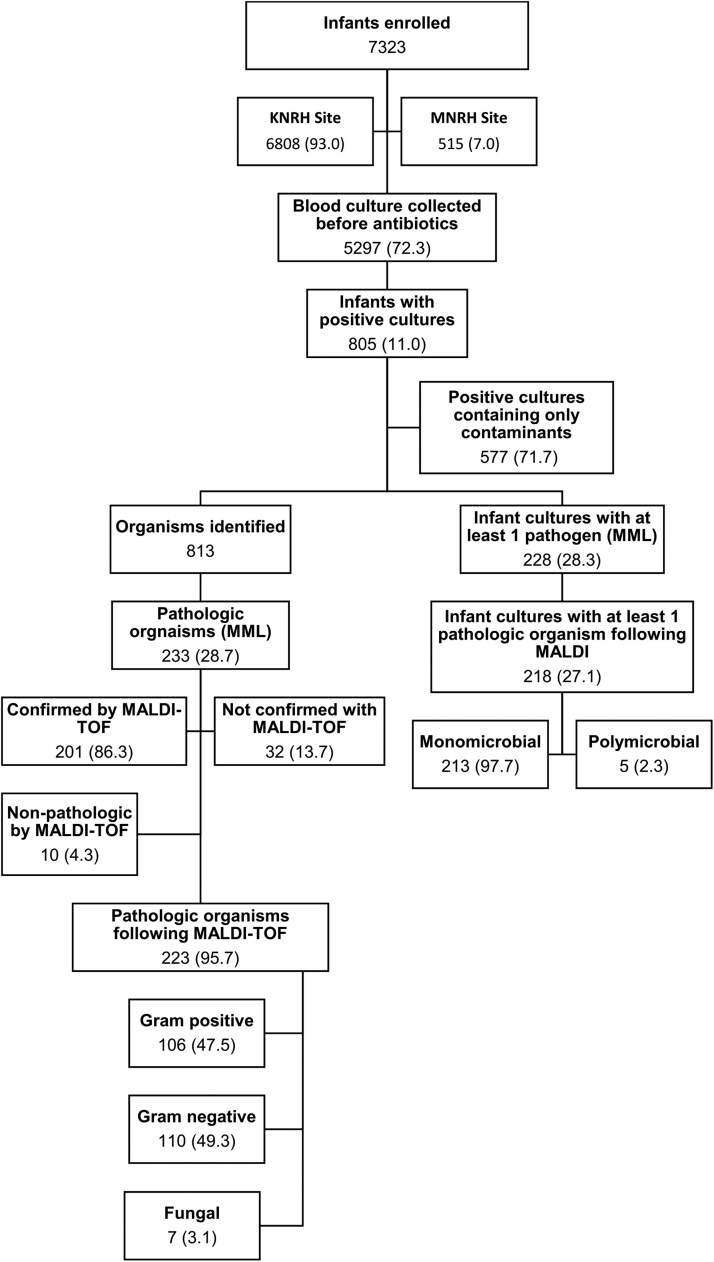
Flow chart of blood culture collection and positivity.

Of the 7323 blood culture samples collected over the study period, 5297 (72.3%) were collected before administration of antibiotics (Figure 1). There was no significant difference in culture positivity rate between those that had a culture collected before antibiotics and those that did not (OR = 1.15, 95% CI, 0.81–1.64).

A total of 805 (11%) blood cultures were positive; 218 (3.0%) from infants with at least one pathogen identified (>1 in 5 cases). Among the 216 bacterial pathogens, Gram-negative infections predominated (110/216, 50.9%). The most frequent Gram-negative organisms were *Escherichia coli* (33, 14.8%), *Acinetobacter* spp. (23, 10.3%) and *Klebsiella pneumoniae* (17, 7.6%), whereas *Streptococcus agalactiae* (30, 13.5%), *Enterococcus* (25, 11.2%), and *Streptococcus viridans* (23, 10.3%) were the most frequent Gram-positive organisms (Table 2). Fungal organisms were identified in 7 (3.1%) of cultures. No cultures had bacteria and fungi co-isolated. Infants with late-onset sepsis were more likely to be infected with a Gram-negative organism than those with early-onset infection (59.4% vs 48.2%).

**Table 2.** Table Showing Identified Bacterial and Fungal Pathogens

Organism	Freq	%
**Bacterial organisms**	216	96.9
**Gram negative**	**110**	(50.9)*
*Escherichia coli*	33	14.8
*Acinetobacter* spp	23	10.3
*Klebsiella pneumoniae*	17	7.6
*Enterobacter* spp	13	5.8
*Pseudomonas* spp	9	4.0
Other Gram negative	15	6.7
**Gram positive**	**106**	(49.1)*
*Streptococcus agalactiae*	30	13.5
*Enterococcus* spp	25	11.2
*Streptococcus viridans*	23	10.3
*Staphylococcus aureus*	17	7.6
*Streptococcus bovis*	6	2.7
Other Gram positive	5	2.2
**Fungal organisms**	**7**	**3.1**
*Candida*	7	3.1
**Total organisms**	**223**	**100**

*Frequency and % of total bacterial organisms.

The most used antibiotic regimens were ampicillin and either amikacin (37.8%) or gentamicin (3.5%) and cefotaxime and amikacin (8.1%) (Supplementary Table 4). Antimicrobial sensitivity testing results were available for 186 (86.1%) bacterial isolates. Antimicrobial resistance to WHO first-line antibiotics (ampicillin/benzylpenicillin and gentamicin/amikacin) was high: 37 (40.7%) of Gram-positive isolates were resistant to one or more first-line antibiotics, whereas 53 (55.8%) of Gram-negative isolates were resistant to both first-line antibiotics (Supplementary Figure 2). Twenty-three infants with suspected meningitis had a lumbar puncture collected; all were negative.

A total of 2563 babies had a nasopharyngeal swab collected (Supplementary Figure 3); 115 (6.1%) were positive with 137 viruses: respiratory syncytial virus (RSV) in 44.5% and rhinovirus in 35.0% (Table 3). Four infants had a positive viral nasopharyngeal swab and a bloodstream infection with either a bacteria (n = 2) or *Candida* spp. (n = 2). A total of 106 (4.9%) infants had swabs that were positive for bacteria with *Haemophilus influenzae* (54.6%) and *Streptococcus pneumoniae* (44.5%) the most common; two of these infants also had a confirmed bloodstream infection with *E coli* (n = 1) or *S aureus* (n = 1). Just over half of infants with positive viral (58.3%) and bacterial (54.7%) swabs had documented respiratory signs such as severe chest indrawing, grunting, and oxygen requirement.

**Table 3.** Viruses and Bacteria Identified From Nasopharyngeal Swabs

Pathogen Identified	Frequency (%)
Viruses (115 positive swabs)	
Respiratory syncytial virus	61 (44.5)
Rhinovirus	48 (35.0)
Influenza A	10 (7.3)
Influenza B	11 (8.0)
Metapneumovirus	3 (2.2)
Adenovirus	2 (1.5)
Parainfluenza	2 (1.5)
Total	**137**
Bacteria (106 positive swabs)	
*Haemophilus influenzae*	65 (54.6)
*Legionella pneumophilia*	1 (0.8)
*Streptococcus pneumonia*	53 (44.5)
Total	**119**

The case fatality was 19.8% in those with confirmed pathogen(s) in blood cultures compared to 11.9% in those with negative blood cultures. Following adjustment for confounding factors, the odds of dying during their admission was 5.22 (95% CI, 3.07–8.87) times higher in infants with greater than three signs of sepsis at admission compared to those with risk factors for sepsis but no clinical signs. HIV-exposed infants had higher odds of dying than unexposed infants (adjusted OR [aOR] = 1.45 [1.07–1.97]). Those that were born prematurely had increased odds of dying (aOR = 1.55 [1.27–1.90]) as did those with a confirmed bloodstream infection (aOR = 2.19 [1.35–3.60]). Infants born by cesarean section had lower odds of dying during their admission than those born vaginally (aOR = 0.69 [0.55–0.86]) (Table 4).

**Table 4.** Logistic Regression Analysis Showing Factors Associated With Inpatient Mortality—Multivariable Analysis (n = 3690)

Risk Factor		Total	Inpatient mortality	aOR	95% CI	*P* Value
Bloodstream infection	Negative/contaminant	5788	687 (11.9)	-	…	
	Pathologic	172	34 (19.8)	2.19	1.35–3.60	.0021
HIV exposure	Unexposed	3739	417 (11.2)			
	Exposed	425	67 (15.8)	1.45	1.07–1.97	.005
Gestational age	Term	2869	301 (10.5)	-	…	
	Preterm	2012	350 (17.4)	1.55	1.27–1.90	<.00001
Age at admission (days)	0–6 d	5099	670 (13.1)	-	…	
	>6 d	315	19 (6.0)	0.42	.25–.71	.0002
Mode of delivery	Vaginal	3287	477 (14.5)	-	…	
	Cesarean	1789	164 (9.2)	0.69	.55–.86	<.00001
Number of signs of sepsis	Risk factors	584	32 (5.5)	-	…	
	1–3 signs	3916	354 (9.0)	1.82	1.07–3.09	
	> 3 signs	1460	335 (23.0)	5.22	3.07–8.87	<.00001

Abbreviations: aOR, adjusted odds ratio; CI, confidence interval.

Of the babies with confirmed bloodstream infections, the case fatality rate during admission from Gram-positive infections was lower than for Gram-negative (11.8% v 27.7%), cOR 0.35 [0.15–0.80]. There was some weak evidence of an association between antibiotic resistance with ≥ 1 first-line antibiotic and death, on unadjusted analysis (OR = 2.89 [1.01–8.29]) (Supplementary Table 6).

## DISCUSSION

Through comprehensive microbiological surveillance conducted in two large Ugandan hospitals, we have defined the current landscape of neonatal sepsis and antimicrobial resistance in this setting. We observed a nearly equal distribution of bloodstream infections due to Gram-positive and Gram-negative pathogens, dominated by *E coli* (14.8%) and *S agalactiae* (13.5%). Viral pathogens were also identified in infants with signs of sepsis, the most common being RSV and rhinovirus. These results provide valuable insights into the potential targets for effective preventive and therapeutic strategies.

Our results contrast with a smaller surveillance study (n = 359 neonates) conducted in Kampala in 2018 in one of the same hospitals as our study (MNRH). This earlier study found that Gram-positive organisms predominated (70%), with a large proportion of cases caused by *S aureus*, but no *S agalactiae* (GBS) [13]. In a more recent, large, international multicenter observational study conducted in a range of hospital settings in LMICs (neonatal sepsis observational cohort study: NeoOBS), a large proportion of Gram-negative organisms (62.9%) were identified, and *S agalactiae* contributed only 3.4% [14]. It is plausible that this difference in GBS isolation rates stems from the unique context of our study, embedded within a rigorously conducted microbiological GBS surveillance study as well as the predominance of early-onset sepsis cases in our cohort (91.4%).

The increasing contribution that Gram-negative organisms make to neonatal bloodstream infections is reflected in many settings [15] and is especially concerning given the increased case-fatality associated with Gram-negative infections, as seen in our study. A large multicenter neonatal sepsis study (BARNARDS) reported similar case fatality rates from Gram-negative neonatal infections in four African settings (21%), as well as similar rates of resistance to first-line antibiotics among Gram-negative infections (60%) [15].

Culture contamination rates were high in our study (577/805) 71.7% of positive cultures; this is higher than the NeoOBS study that identified 16.9%. However, they classified Coagulase-negative staphylococci (CoNS) as presumed pathogens in their study, whereas we have identified them as probable contaminants. CoNS accounted for 326/590 (55.3%) of all probable contaminants identified in our study. CoNS are normal part of the human microbiome; however, the significance of *Staphylococcus haemolyticus* as a neonatal pathogen has received increased recognition in recent years [16]. A comparison of commensal and clinical *S haemolyticus* isolates found clear differences in genotypes and genetic determinants associated with success in the hospital environment as well as pathogenicity; they may therefore be an important cause of nosocomial infections [17]. Studies have shown that neonates born preterm and very low birth weight <1500 g may have a greater risk of contracting CoNS, particularly as a late-onset infection [18]. In our study, 34 babies that grew CoNS in their blood cultures were born low birthweight and 70 had a late-onset infection, CoNS may be a pathogenic organism in a number of these cases. Future work will consider whole-genome sequencing of *S haemolyticus* to further characterize these isolates.

In keeping with recent point-prevalence surveys, our study shows divergence from WHO-recommended empiric, first-line antibiotic regimens [19]. Most infants received regimens made up of WHO access antibiotics and 26% were given at least 1 of the watch-list antibiotics, most frequently cefotaxime. It was also common for infants to receive both a third-generation cephalosporin and an aminoglycoside concurrently. A quarter of infants (24.7%) with a bloodstream infection demonstrating resistance to at least 1 first-line antibiotic in our study died during their admission compared to 10.2% of infants with sensitive infections (Supplementary Table 6). Although AMR has been demonstrated as an independent predictor of mortality in adults with bloodstream infections in LMIC settings, increasing mortality by up to 58% [20], information on neonates and infants is scarce, relying largely on data from retrospective epidemiological studies [21]. Unfortunately, the numbers with complete antimicrobial therapy and resistance information were too small in our study to assess the independent impact of antimicrobial resistance on mortality in adjusted analyses; such data are essential to understand the impact of antimicrobial resistance on infant outcomes.

We have identified RSV (44.5%), *H influenzae* (54.6%), and *S pneumoniae* (44.5%) as the most common viral and bacterial pathogens detected on NPS with just more than half of these infants exhibiting respiratory signs. Differentiating from other causes of respiratory distress in neonates is challenging lower respiratory tract infections. Over the past century, findings of etiological studies of pneumonia have evolved from sole detection of bacteria to the recognition of the importance of viral infections and associated changes in methodological approaches [22]. Blood cultures are an insensitive test for determining the etiology of bacterial pneumonia, and past studies have reported varied estimates of the prevalence of bacteremia between 1% and 10% [23, 24]. Upper respiratory tract specimens such as nasopharyngeal swabs can be helpful for inferring the cause of lower respiratory tract infections, or viral sepsis due to ease of collection; however, a positive test may represent commensal colonization of bacteria or infection limited to the upper respiratory tract [22]. Better diagnostics for lower respiratory tract infections continue to be required to understand their etiology and implement effective therapeutics.

### Limitations

There are some limitations to this study. Blood cultures were obtained from 67% (6808/10 108) of infants admitted with suspected sepsis at KNRH. Unfortunately, denominator data for MNRH were not accessible. Non-collection of blood cultures at both hospitals was due to random factors such as stock-outs of blood culture bottles and staff shortages. It is likely, therefore, that there are no important differences between those that did and did not receive a blood culture that would introduce bias into our sampling; however, we have not been able to investigate this. Data collection relied on details documented in the infants’ clinical records that were not always comprehensively recorded, resulting in some missing data. Training was given to staff on lumbar puncture collection and resources were provided for this; however, there were cultural barriers locally toward acceptance of lumbar puncture by caregivers, and therefore uptake was low in our study. Finally, our study was based in two tertiary referral hospital sites in an urban center and may not be reflective of the epidemiology of neonatal infections in the broader community. Our results may therefore overestimate the burden of Gram-negative infections and AMR [5].

## CONCLUSIONS

In conclusion, our study sheds light on the complex etiology of infection in a large infant cohort in Uganda, highlighting critical findings that underscore the urgency of addressing this public health concern. The overall inpatient mortality rate of 12.1% is a stark reminder of the severity of neonatal sepsis in this region, with a 27.7% mortality rate observed among infants with Gram-negative bloodstream infections. In a later paper in this supplement by Sadoo et al, we demonstrate high rates of moderate to severe neurodevelopmental impairment among infant survivors of GBS infection from this cohort; although survival is of critical importance, attention must also be given to understanding long-term development following infections. Moving forward, concerted efforts must be made to combat the growing threat of neonatal sepsis in Uganda. This involves not only the enhancement of diagnostic and treatment protocols but also a broader public health approach to curb AMR and provide preventive measures through vaccination.

## Supplementary Data

Supplementary materials are available at *Open Forum Infectious Diseases* online. Consisting of data provided by the authors to benefit the reader, the posted materials are not copyedited and are the sole responsibility of the authors, so questions or comments should be addressed to the corresponding author.

## Supplementary Material

ofae629_Supplementary_Data

## Notes

***Acknowledgments.*** The authors thank the staff at KNRH, MNRH, and MUCML, as well as all the families and children involved in the study. They recognize their esteemed lab manager, Hannington Tasimwa, who tragically lost his life to COVID-19 during the course of this research. His dedication, expertise, and unwavering support significantly contributed to the success of this project. The authors are deeply grateful for this enduring impact on our work and will forever cherish his memory. The complete PROGRESS Study Author Group comprises Abdelmajid Djennad, Agnes Nyamaizi, Agnes Ssali, Alexander Amone, Amusa Wamawobe, Annettee Nakimuli, Caitlin Farley, Carol Nanyunja, Christine Najuka, Cleophas Komugisha, Dan R. Shelley, Edward A. R. Portal, Ellie Duckworth, Emilie Karafillakis, Geraldine O'Hara, Godfrey Matovu, Hannah G. Davies, Janet Seeley, Joseph Peacock, Juliet Nsimire Sendagala, Katie Cowie, Kirsty Le Doare, Konstantinos Karampatsas, Lauren Hookham, Madeleine Cochet, Margaret Sewegaba, Mary Kyohere, Maxensia Owor, Melanie Etti, Merryn Voysey, Moses Musooko, Musa Sekikubo, Owen B. Spiller, Patience Atuhaire, Paul T. Heath, Philippa Musoke, Phiona Nalubega, Pooja Ravji, Richard Katungye, Ritah Namugumya, Rosalin Parks, Rose Azuba, Sam Kipyeko, Simon Beach, Stephen Bentley, Tim Old, Tobius Mutabazi, Valerie Tusubira and Vicki Chalker.

***Author contributions.*** K.L.D., M.S., and P.M. conceived of the study design and methodology. M.K., H.G.D., V.T., A.A., L.H., P.R., M.E., and J.P. were responsible for conduct of the study and oversight of data collection activities. Data management activities were managed by V.T. and C.K. Laboratory analysis was overseen and undertaken by O.B.S., A.W., L.H., M.E., C.F., D.R.S., and P.R. K.L.D., H.G.D., M.K., V.T., J.N.S., and M.V. analyzed and interpreted the data. H.G.D., M.K., and V.T. drafted the manuscript. All authors contributed to further revisions of the manuscript and all authors reviewed and approved the final manuscript.

***Data availability statement.*** The full dataset for the PROGRESS study is available at doi:10.24376/rd.sgul.24101115.v1 [24].

***Ethics statement.*** The study has been approved by the Ugandan National Council for Science and Technology (UNCST; ref. HS 2496), Makerere University School of Medicine Research & Ethics Committee (SOMREC; 2018–130), and St George's, University of London (SGUL REC ref 2020.0024) who sponsored the study. The protocol was published in Gates Open Research in June 2020 [10].

***Financial support*.** This work was supported by the Bill and Melinda Gates Foundation [OPP1189053]. This work was also supported by the National Institute for Healthcare Research for the qualitative research [16/136/46], and sponsored by St George’s, University of London (SGUL) R&D Protocol Code Number: 17.0018. The funder of the study had no role in study design, data collection, data analysis, data interpretation, writing of the manuscript, or decision to submit the manuscript for publication. The funders had no access to the dataset of this study.

***Supplement sponsorship***. This article appears as part of the supplement “Neonatal and Young Infant Infection in Uganda: the PROGRESS Study,” sponsored by St George's, University of London.
