# Supplementary material for: Etiology and Antimicrobial Resistance of Culture-Positive Infections in Ugandan Infants: A Cohort Study of 7000 Neonates and Infants
Source: Open Forum Infect Dis. 2025 Mar 10;11(Suppl 3):S157–64. doi: 10.1093/ofid/ofae629 (PMC11891137; doi:10.1093/ofid/ofae629)
Supplement: ofae629_Supplementary_Data [file ofae629_supplementary_data.zip › Sepsis_Supplementary.docx]

Supplementary Figure 1 - Study sites and participant recruitment for PROGRESS supplement studies

^#^ Includes HIV exposed and unexposed infants and infants form the birth cohort.

Supplementary Figure 2- Antimicrobial resistance results for gram positive and gram-negative organisms

**Two organisms demonstrated intermediate resistance to benzylpenicillin and sensitivity to gentamicin*

Supplementary Table 1 - Table of all organisms considered as contaminants.

| **Organisms Identified - Blood Culture** | **Freq.** | **Proportion** |
| --- | --- | --- |
| *Coagulase Negative Staphylococcus (CoNS)* | 326 | 55.3 |
| *Micrococcus spp* | 120 | 20.3 |
| *Bacillus* | 89 | 15.1 |
| *Corynebacterium* | 44 | 7.5 |
| *Aerococcus viridans* | 2 | 0.3 |
| *Gram positive cocci in clusters* | 1 | 0.2 |
| *Proteus mirabilis* | 1 | 0.2 |
| *Rhodococcus species* | 5 | 0.85 |
| *Unidentified fungus* | 2 | 0.3 |
| *Total* | 590 | 100 |

Supplementary Table 2 - Maximum appropriate blood volume to be drawn for study procedures according to weight of baby*

| **Weight of baby (kilograms)** | **Maximum volume of blood in single draw* (millilitres)** |
| --- | --- |
| **4** | 3.2 |
| **3** | 2.4 |
| **2** | 1.6 |
| **1.5** | 1.2 |
| **1.3** | 1.0 |
| **1.0** | 0.8 |
| **0.75** | 0.6 |
| **0.5** | 0.4 |

*Adapted from: ETHICAL CONSIDERATIONS FOR CLINICAL TRIALS ON MEDICINAL PRODUCTS CONDUCTED WITH THE PAEDIATRIC POPULATION Recommendations of the ad hoc group for the development of implementing guidelines for Directive 2001/20/EC relating to good clinical practice in the conduct of clinical trials on medicinal products for human use.

Supplementary Table 3 - All pathogenic organisms identified

| **Organism identified** | **Freq.** | **%** |
| --- | --- | --- |
| Acinetobacter insolitus | 1 | 0.4 |
| Achromobacter xylosoxidans | 1 | 0.4 |
| Acinetobacter baumannii | 10 | 4.5 |
| Acinetobacter colistiniresistens | 2 | 0.9 |
| Acinetobacter haemolyticus | 2 | 0.9 |
| Acinetobacter nosocomialis | 3 | 1.3 |
| Acinetobacter seifertii | 1 | 0.4 |
| Acinetobacter spp | 5 | 2.2 |
| Candida | 7 | 3.1 |
| Citrobacter freundii | 1 | 0.4 |
| Citrobacter spp | 3 | 1.3 |
| Enterobacter bugandensis | 3 | 1.3 |
| Enterobacter cloacae | 2 | 0.9 |
| Enterobacter hormaechei | 1 | 0.4 |
| Enterobacter kobei | 2 | 0.9 |
| Enterobacter roggenkampii | 5 | 2.2 |
| Enterococcus faecalis | 11 | 4.9 |
| Enterococcus faecium | 9 | 4.0 |
| Enterococcus spp | 5 | 2.2 |
| Escherichia coli | 33 | 14.8 |
| Klebsiella pneumoniae | 17 | 7.6 |
| Moraxella spp | 2 | 0.9 |
| Neisseria Meningitidis | 1 | 0.4 |
| Pseudomonas aeruginosa | 9 | 4.0 |
| Ralstonia pickettii | 1 | 0.4 |
| Sphingomonas paucimobilis | 2 | 0.9 |
| Staphylococcus aureus | 17 | 7.6 |
| Stenotrophomonas maltophilia | 3 | 1.3 |
| Streptococcus agalactiae | 30 | 13.5 |
| Streptococcus anginosus | 2 | 0.9 |
| Streptococcus gallolyticus | 5 | 2.2 |
| Streptococcus gordonii | 1 | 0.4 |
| Streptococcus infantarius | 1 | 0.4 |
| Streptococcus mitis oralis | 11 | 4.9 |
| Streptococcus parasanguinis | 3 | 1.3 |
| Streptococcus pneumoniae | 2 | 0.9 |
| Streptococcus pyogenes | 3 | 1.3 |
| Streptococcus salivarius | 1 | 0.4 |
| Streptococcus sanguinis | 2 | 0.9 |
| Viridans Streptococcus | 3 | 1.3 |
| Total* | 223 | 100.00 |

*223 organisms from 218 blood cultures (5 polymicrobial cultures)

Supplementary Table 4 - Antimicrobial regimens administered to infants with signs or risk-factors for sepsis (n=4912).

| **Antimicrobial regimens (n=4912)** | **Freq (%)** |
| --- | --- |
| Ampicillin | 507 (6.9) |
| Ampicillin & gentamicin | 254 (3.5) |
| Ampicillin & amikacin | 2771 (37.8) |
| Ampicillin & cefotaxime | 92 (1.3) |
| Cefotaxime & amikacin | 593 (8.1) |
| Ampicillin, amikacin & cefotaxime | 110 (1.5) |
| Cefotaxime | 191 (2.6) |
| Other combination | 392 (5.4) |
| **Antimicrobial therapy missing** | **2411 (32.9)** |

Supplementary Table 5 - Antimicrobial resistance patters of gram positive and negative organisms.

|  | **Sensitive** | **Intermediate** | **Resistant** | **Total** |
| --- | --- | --- | --- | --- |
| **Gram Positive (n=106)** |  |  |  |  |
| Benzylpenicillin | 57 (62.6) | 2 (2.2) | 32 (35.2) | 91 (85.8) |
| Gentamicin | 71 (78.0) | 0 (0) | 20 (22.0) | 90 (85.8) |
| **Gram Negative (n=110)** |  |  |  |  |
| Ampicillin | 88 (92.6) | 0 (0) | 7 (7.4) | 95 (86.4) |
| Gentamicin | 41 (43.2) | 0 (0) | 54 (56.8) | 95 (86.4) |
| Ceftazidime | 28 (29.5) | 20 (21.1) | 47 (49.5) | 95 (86.4) |
| Amikacin | 80 (85.1) | 0 (0) | 14 (14.9) | 94 (85.5) |

Supplementary Table 6 - Factors associated with case fatality in babies with bloodstream infections – univariate analysis.

| **Risk Factor** |  | **Total** | **Died** | **cOR** | **95% Cl** | **P value** |
| --- | --- | --- | --- | --- | --- | --- |
| **Baby Sex**  (n=152) | Male | 76 | 17 (22.4) | - |  |  |
|  | Female | 76 | 15 (19.7) | 0.85 | 0.39-1.87 | 0.69 |
| **HIV Exposure** (n=129) | Unexposed | 120 | 23 (19.2) | - |  |  |
|  | Exposed | 9 | 3 (33.3) | 2.11 | 0.48-9.17 | 0.31 |
| **Birthweight**  (n=110) | ≥2500g | 61 | 11 (18.3) | - |  |  |
|  | <2500g | 49 | 15 (30.61) | 2.01 | 0.81-4.96 | 0.12 |
| **Age at admission**  (n=161) | 0-6 days | 136 | 27 (19.9) | - |  |  |
|  | >6 days | 25 | 6 (24.0) | 1.27 | 0.46-3.51 | 0.64 |
| **Mode of delivery**  (n=152) | Vaginal | 108 | 22 (20.3) | - |  |  |
|  | Caesarean | 44 | 8 (18.2) | 0.87 | 0.35-2.14 | 0.76 |
| **Signs of sepsis**  (n=172) | Risk factors only | 13 | 2 (15.4) | - |  |  |
|  | 1-3 signs | 111 | 17 (15.3) | 1.0 | 0.20-4.92 | 0.04 |
|  | > 3 signs | 48 | 15 (31.3) | 2.5 | 0.48-13.10 |  |
| **Gestational age at birth**  (n=142) | Term | 79 | 12 (15.2) | - |  |  |
|  | Preterm | 63 | 18 (28.6) | 2.23 | 0.96-5.15 | 0.05 |
| **Gram Status**  (n=168) | Gram negative | 83 | 23 (27.7) | - |  |  |
|  | Gram positive | 85 | 10 (11.8) | 0.35 | 0.15-0.80 | 0.0095 |
| **Antimicrobial resistance**  (n=184)* | No resistance | 58 | 5 (10.2) |  |  |  |
|  | ≥ 1 first line antibiotic | 126 | 24 (24.7) | 2.89 | 1.01-8.29 | 0.038 |

**Two organisms demonstrated intermediate resistance to benzylpenicillin and sensitivity to gentamicin*

Supplementary Figure 3- Flow chart of nasopharyngeal swab collection and positivity
